# Impact of fungal biliary colonization on postoperative outcomes following pancreaticoduodenectomy: A systematic review

**DOI:** 10.12669/pjms.42.(11AASC).15653

**Published:** 2026-04

**Authors:** Nargis Maqbool, Rameesha Ahmad, Saleema Begum, M. Rizwan Khan

**Affiliations:** 1 Dr. Nargis Maqbool, Department of Surgery, Aga Khan University Hospital, Karachi, Pakistan; 2 Dr. Rameesha Ahmad, Medical College, Aga Khan University Hospital, Karachi, Pakistan; 3 Dr. Saleema Begum, Department of Surgery, Aga Khan University Hospital, Karachi, Pakistan; 4 Dr. Muhammad Rizwan Khan, Department of Surgery, Aga Khan University Hospital, Karachi, Pakistan

**Keywords:** Candida, Fungal Colonization, Pancreaticoduodenectomy, Preoperative Biliary Drainage, Postoperative Complications, Surgical Site Infection

## Abstract

**Background and Objective:**

Pancreaticoduodenectomy (PD) is associated with substantial postoperative morbidity, with infectious complications representing a major contributor. Preoperative biliary drainage (PBD) often leads to bile contamination, but the clinical significance of fungal colonization, particularly by Candida species, remains unclear. This systematic review aimed to evaluate the impact of fungal biliary colonization on postoperative outcomes following PD.

**Methodology:**

A systematic search of PubMed, Embase, Scopus, and Cochrane CENTRAL was performed up to October 15, 2025, for studies reporting microbiologically confirmed biliary fungal colonization in adult PD patients. Eligible studies included cohort and case-control designs reporting postoperative outcomes. Data extraction focused on infectious complications, clinically relevant postoperative pancreatic fistula (CR-POPF), overall morbidity, mortality, length of hospital stay, and antifungal therapy. Risk of bias was assessed using the Newcastle-Ottawa Scale.

**Results:**

Nine studies (seven retrospective cohorts, one prospective observational, one multicenter retrospective) including 2,626 patients were included and qualitatively synthesized. Fungal biliary colonization occurred predominantly in PBD patients, with Candida species most frequently isolated. Colonization rates ranged from 2.5% to 43%. Evidence regarding postoperative outcomes was heterogeneous: while some studies identified fungal colonization as a risk factor for surgical site infection (SSI), most studies found no independent association with SSI, CR-POPF, overall morbidity, or mortality after accounting for bacterial contamination and PBD. Antifungal therapy was used inconsistently, with limited evidence suggesting potential benefit in selected high-risk patients.

**Conclusions:**

Fungal biliary colonization is common in patients undergoing PBD, but current evidence suggests it is primarily a marker of biliary manipulation and bacterial co-contamination rather than an independent driver of postoperative morbidity or mortality. Routine antifungal prophylaxis is not supported, although targeted therapy may benefit high-risk individuals. Prospective multicenter studies with standardized microbiological assessment are needed to clarify the clinical significance of fungal colonization in PD.

**PROSPERO) (Registration ID:** CRD420251178848.

## INTRODUCTION

Pancreaticoduodenectomy (PD), or the Whipple procedure, remains the principal curative operation for malignancies of the pancreatic head, distal bile duct and periampullary region.^1^ Despite major advances in perioperative care, PD continues to carry substantial morbidity, with postoperative complications rates reported between 40-58%.^2^ Infectious complications, particularly surgical site infections (SSI), intra-abdominal abscess, sepsis and clinically relevant postoperative pancreatic fistula (CR-POPF) represents major contributors to morbidity, prolonged hospitalization and mortality following PD.^3^,^4^

Preoperative biliary drainage (PBD) is widely used to relieve obstructive jaundice,^5^ particularly in patients with cholangitis or when surgery is delayed due to medical optimization, staging workup or neoadjuvant therapy.^6^ However, PBD is associated with bile contamination in up to 85-90% of cases.^7^ While the clinical impact of bacterial contamination has been well studied, demonstrating associations with higher rates of infectious morbidity and POPF^8^, the clinical impact of fungal biliary colonization, particularly by Candida species, remains poorly understood.^9^ Reported rates of fungal colonization range from 11.7% to 29%, with nearly all cases occurring in PBD group.^10^,^11^

The impact of this fungal colonization on postoperative outcomes remains uncertain. Some studies suggest that fungal contamination does not independently increase postoperative infectious complications when compared with bacterial contamination alone.^12^ In contrast, other reports indicate that Candida species in biliary or peripancreatic samples may be associated with more severe postoperative morbidity including hemorrhagic complications secondary to POPF, higher rates of intra-abdominal infection and prolonged hospital stay.^13^ These conflicting findings underscore an important gap in the current understanding of whether fungal biliary colonization is a true pathogenic factor, a marker of severe biliary obstruction or prolonged stenting, or simply a colonization phenomenon without direct clinical consequence.

Importantly, no consensus exists on whether the detection of fungi in bile warrants changes in perioperative antimicrobial strategies. Practices vary widely, with some institutions administering targeted antifungal prophylaxis while others treating fungal colonization as clinically insignificant.^14^ Consequently, clinicians lack guidance on whether fungal organisms meaningfully influence postoperative outcomes or whether routine antifungal coverage is justified. As a result, there is currently no consensus regarding screening, interpretation, or management of fungal colonization in the biliary tract.

This systematic review aimed to synthesize the available evidence on the impact of fungal biliary colonization on postoperative outcomes following pancreaticoduodenectomy. By consolidating current evidence, this review seeks to inform perioperative management strategies and identify gaps for future research in this evolving field.

## METHODOLOGY

This systematic review adhered to the 2020 Preferred Reporting Items for Systematic Review and Meta-Analyses (PRISMA) guidelines and was prospectively registered with the International Prospective Register for Systematic Reviews (PROSPERO) (Registration ID: CRD420251178848). The PRISMA Checklist is included in Supplementary Section 1.

A pre-defined protocol was developed prior to specifying the research question, eligibility criteria, and planned methods of synthesis. A comprehensive systematic literature search was performed on electronic databases including PubMed, Embase, Scopus, and Cochrane CENTRAL from database inception to October 15, 2025, using MeSH terms and keywords related to fungal colonization, biliary microbiology, and pancreaticoduodenectomy. The search strategy used for the above-mentioned databases has been included in Supplementary Section 2.

### Eligibility criteria and Study selection:

Studies were eligible if they involved adult patients ((≥18 years) undergoing pancreaticoduodenectomy, reported biliary fungal colonization confirmed by microbiological culture, and described impact of fungal colonization on postoperative outcomes. Eligible study designs included cohort studies, case-control studies, and randomized controlled trials. Studies were excluded if they were case reports or small case series with fewer than 10 patients, if they focused solely on non-biliary fungal infections, or if postoperative outcomes were not reported. Non-original research, such as expert opinion, editorials, and conference abstracts without full text, was also excluded.

**Fig.1:**
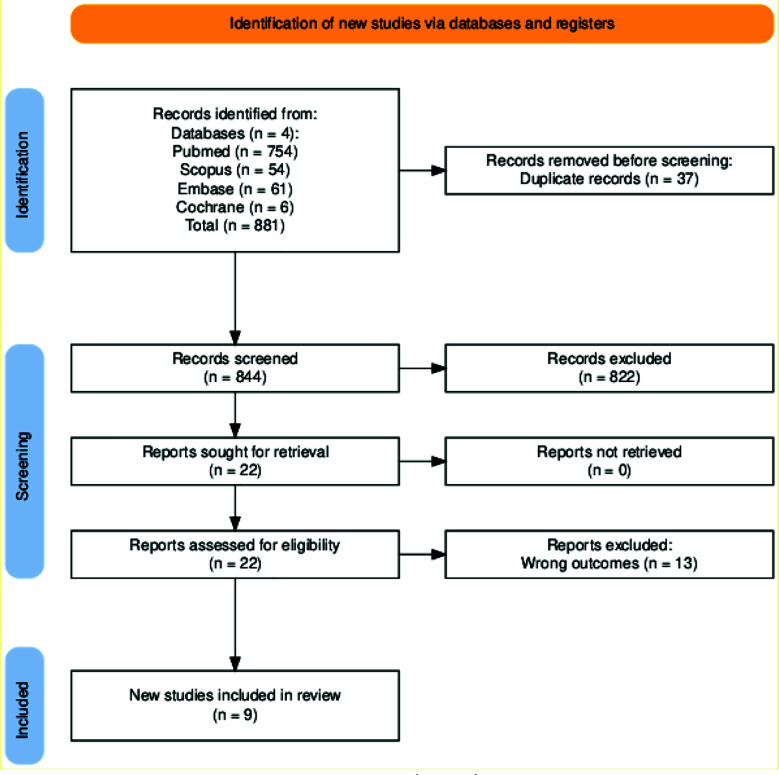
PRISMA Flow Diagram.

Two reviewers (N.M. and R.A.) independently screened titles and abstracts, followed by full-text review of relevant articles. Disagreements at either stage were resolved through consensus discussion or adjudication by a third reviewer. Study selection is illustrated in the PRISMA flow diagram (Fig.1).

### Data extraction:

Data extraction was performed using a standardized form, including study characteristics, patient demographics, indication for surgery, PBD details, bile sampling method and timing, type of fungal organisms isolated, postoperative outcomes (primary and secondary), use of antifungal therapy and key conclusions of the study.

The primary outcomes of interest for this review were postoperative infectious complications (SSI, intra-abdominal abscess, sepsis), clinically relevant POPF grade B/C, overall morbidity and 30-day or 90-day mortality. Secondary outcomes included length of hospital stay, reoperation, ICU admission, and use and impact of antifungal therapy.

Given heterogeneity, a qualitative synthesis was performed. Outcomes were summarized using the effect measures reported in the individual studies (proportions, means or medians, odds ratios or hazard ratios where available.

### Risk of bias assessment:

Risk of bias for observational studies was assessed using the Newcastle-Ottawa Scale (NOS) across selection, comparability, and outcome domains. Two reviewers independently assessed quality, with consensus resolution of discrepancies (Table-I).

**Table-I:** Quality Assessment using Newcastle–Ottawa Scale (NOS)

Study (Author, year)	Study Design	Selection (0- 4)	Comparability (0-2)	Outcome (0-3)	Total (0-9)	Risk of bias
Kato H et al. (2018)	Prospective observational	4	2	3	9	Low
Maatman TK et al. (2019)	Retrospective observational	3	0	1	4	High
Nakamura K et al. (2020)	Retrospective cohort	3	1	2	6	Moderate
Tortajada P et al. (2020)	Multicenter retrospective cohort	3	1	2	6	Moderate
Lin YJ et al. (2021)	Retrospective cohort	4	1	2	7	Low
Krueger CM et al. (2022)	Retrospective cohort	3	1	2	6	Moderate
Camps-Lasa J et al. (2024)	Retrospective cohort	4	2	2	8	Low
Giannone F et al. (2025)	Retrospective cohort	3	1	2	6	Moderate
Yang Y et al. (2025)	Retrospective cohort	3	1	2	6	Moderate

**Table-II:** Study Characteristics

Author (Year)	Study Design	Study Period	Sample Size (n)	PBD	Population & Stratification	Bile Sampling	Fungal Species Reported	Primary and Secondary Outcomes Reported	Main Outcomes Relevant to Fungal Colonization
Kato H et al. (2018)	Prospective observational	2014–2016	51	31.37%	Stratified by biliary candidiasis (BC)	Postoperative biliary stent sampling on POD 3/7/14	Biliary candidiasis 43.1%; Candida albicans: the most frequent 82%	Primary: SSI; CR-POPF	SSI significantly higher in BC group (71 % vs 6.8 %, p = 0.005); BC independent predictor of SSI (p=0.002); Among Candida species, C. Glabrata associated with higher SSI incidence (p=0.032)
Maatman TK et al. (2019)	Retrospective observational	2015–2017	162	60%	Bacteriobilia + vs –	Intraoperative bile culture	Yeast in 19%, not speciated	Primary: SSI type, cholangitis CR-POPF, 30 or 90-day mortality	Bacteriobilia associated with higher SSI; no fungal impact detected.
Nakamura K et al. (2020)	Retrospective cohort	2013–2018	209	48.80%	PD patients; intraop bile + POD 4 drain culture	Intraoperative and postoperative cultures	Candida, not speciated	Primary: CR-POPF	SSI associated with positive bile cultures; Candida not associated with CR-POPF (p=0.34); fungal impact on other outcomes not analyzed
Tortajada P et al. (2020)	Multicenter retrospective cohort	2012–2018	224	100%	B+ (n=154) vs BF+ n=52)	Intraoperative bile culture	Fungal contamination in 25%. Candida albicans most frequent pathogen isolated 44%	Primary: Overall infectious complications, intra-abdominal collection, sepsis, cholangitis, overall morbidity, CR-POPF Grade B/C. Secondary: reoperation, 90-day mortality duration, microbiology	Fungal biliary contamination not associated with higher postoperative complication rates compared with bacterial contamination alone.
Lin YJ et al. (2021)	Retrospective cohort	2007–2019	539	41.80%	Bile contamination + (433) vs – (106)	Intraoperative swab after CBD transection	Candida (not speciated)	Primary: Intra-abdominal abscess (iAA), SSI, CR-POPF, mortality Secondary outcomes: length of hospital stay	Candida associated with iAA (OR=4.994; 95% CI:1.837-13.572; p=0.002); regression analysis confirmed independent risk (OR=4.666; 95% CI: 1.677–12.981; p = 0.003); SSI not clearly associated
Krueger CM et al. (2022)	Retrospective cohort	2009-2019	426	49.80%	stratified by PBD, bacteriobilia (BB) and fungibilia (FB)	Intraoperative bile fluid smear after CBD transection	Candida spp, not speciated	Primary: Incidence of isolated infectious complications (iiC) defined as postoperative infections without intra-abdominal focus (e.g., SSI, pneumonia, cholangitis, infection of unknown origin); Secondary: length of postoperative stay	iiC occurred in 22% (93/426), of which 14% had candidiasis; FB + and BB + associated with SSI; median hospital-stay longer in iiC patients (25d vs. 15d; p<0.01)
Camps-Lasa J et al. (2024)	Retrospective single-center	2014–2021	127 (NP 84; P 43)	34%	PD patients with/without PBD	Intraoperative culture after CHD transection	Candida in P group (16.3 %)	Primary: SSI	Positive bile culture significantly associated with SSI; Candida did not increase SSI (p=0.071); antifungal use not part of protocol
Giannone F et al. (2025)	Retrospective cohort	2014–2022	205	62%	PD subset; stratified by antimicrobial strategy and fungal contamination n=66 vs no fungal contamination n=109	Intraoperative bile culture with adapted prophylaxis	Fungal contamination in 37.7% Among which Candida albicans was the most frequent 50 (75.8%)	Primary: overall infectious complications, SSI, CR-POPF, overall morbidity and mortality Secondary: preop ICU admission, impact of antifungal therapy	Fungal contamination not associated with postoperative infectious complications and CR-POPF, associated with higher preoperative ICU admissions (p=0.005). Targeted antifungal therapy improved outcomes: reduced CR-POPF (p < 0.001), urinary infections (p < 0.001), pneumoniae (p = 0.012), and acute respiratory failure (p = 0.040).
Yang Y et al. (2025)	Retrospective cohort	2018–2024	323	59.10%	PD patients with vs without PBD	Intraoperative bile culture after transection of bile duct	Fungus (2.5% %) mostly in PBD group; unspecified	Primary: Organ/space SSI	Fungal contamination mostly in PBD group; Fungus not associated with organ/space SSI (p=0.097)

***Abbreviations:*** PBD: Preoperative biliary drainage, n: Sample size, BC: biliary candidiasis, POD: Post-operative day, SSI: Surgical site infection, CR-POPF: Clinically relevant post-operative pancreatic fistula, PD: Pancreaticoduodenectomy, B+ or BB+: Bacteriobilia, BF+ or FB+: Fungibilia, iAA: Intra-abdominal abscess, OR: Odds Ratio, iiC: isolated infectious complications, d: days, P group: patients with PBD, NP group: patients without PBD, CHD: common hepatic duct.

**Table-III:** Summary of Outcomes Relevant to Fungal Colonization

Author (Year)	Infectious morbidity (SSI, iAA, Sepsis)	CR-POPF	Overall morbidity	30 or 90 day Mortality	Length of hospital stay	Reoperation	Readmission	ICU admission	Antifungal therapy
Kato H et al. (2018)	SSI incidence was 27.5%. SSI significantly higher in BC (71% vs 6.8%; p=0.005); BC independent predictor of SSI	Reported but not analyzed	Not reported	Not reported	Not reported	Not reported	Not reported	Not reported	Antifungals not routinely used; no effect assessed.; preoperative antibiotics significant for BC development
Maatman TK et al. (2019)	No fungal impact detected; focus on bacteriobilia	Not associated	Not associated	No association	Not reported	Not reported	Not reported	Not reported	Not reported
Nakamura K et al. (2020)	SSI significantly associated with positive bile cultures; fungal impact not seen	Candida not associated	Overall complications higher with bile contamination; fungal impact not studied	Not reported	Not reported	Not reported	Not reported	Not reporter	Not discussed
Tortajada P et al. (2020)	No independent association; fungal contamination did not increase infectious complications, intraabdominal abscess, sepsis, cholangitis compared with bacterial contamination	No association	No increase compared with bacterial contamination alone	90- day mortality: No significant difference	Not reported	No significant difference	Not reported	Not reported	Antifungals not routinely used, no effect identified
Lin YJ et al. (2021)	Candida associated with iAA (OR = 4.994; 95% CI: 1.837–13.572; p = 0.002). SSI: no association (fungal effect not clear)	No clear association	Higher with bile contamination; fungal effect unclear	Not reported	Prolonged with bile contamination	Not reported	Not reported	Not reported	Ineffective therapy significantly associated with IAA (OR = 2.725; 95% CI: 1.840–4.036; *p* < 0.001); antifungal use not reported
Krueger CM et al. (2022)	iiC: FB+ vs FB -: 34/392; SSI: 6(18%)/18 (5%); Candida significantly associated with SSI (p<0.01); cholangitis: no impact	Not reported	Overall infectious complications higher in FB+ group	Not reported	Longer with iiC, fungi not isolated as cause	Not reported	Not reported	Not reported	Antifungal use not standardized; impact unclear
Camps-Lasa J et al. (2024)	Positive bile culture associated with SSI; Candida did not increase SSI (p=0.71)	Not reported	Not reported	Not reported	Not reported	Not reported	Not reported	Not reported	Antifungal use not part of protocol; no effect assessed
Giannone F et al. (2025)	Fungal contamination not associated with overall infectious complications, SSI and bacteremia	Fungal contamination was not associated with CR-POPF	Not associated with overall or major complications	Not associated with postoperative death	Shorted after adapted prophylaxis	Not reported	Not reported	Fungal contamination associated with more preoperative ICU admissions (p=0.005)	Targeted antifungal therapy associated with improved outcomes. CR-POPF reduced in whole cohort (p < 0.001)
Yang Y et al. (2025)	Higher SSI with positive bile culture; fungus not associated with organ/space SSI (p=0.097)	Not associated with positive bile culture	Not significantly associated with positive bile culture	Not reported	Not reported	Not reported	Not reported	Not reported	Antifungal therapy not routinely used

***Abbreviations:*** SSI: Surgical site infections, iAA: intra-abdominal abscess, CR-POPF: Clinically relevant post-operative pancreatic fistula, BC: Biliary candidiasis, OR: Odds ratio, iiC: isolated infectious complications

## RESULTS

A total of 844 records were screened, of which nine studies fulfilled the eligibility criteria. These consisted of seven retrospective cohorts, one retrospective observational study, and one prospective observational study published between 2016 and 2025. All studies obtained intraoperative and/or postoperative bile cultures, though sampling techniques varied considerably, including swabs from the transected common bile duct, cultures from hepatic duct stump, and postoperative biliary stent or drain cultures.

Candida species were the predominant fungi isolated. Reported fungal biliary colonization rates ranged from 2.5% to 43%, across different sampling strategies, with a consistent association with PBD.

### Association Between Fungal Biliary Colonization and Postoperative Outcomes:

### Surgical Site Infection (SSI) and Organ/Space Infection:

Evidence linking biliary fungal contamination to SSI demonstrated substantial heterogeneity across studies. The only prospective study, Kato et al.^17^ (2018) identified biliary candidiasis as a strong and independent risk factor for postoperative SSI (71% vs 7%, p = 0.005). Similarly, Krueger et al.^18^ (2022) reported higher SSI rates among patients with fungibilia compared to those without (18% vs 5%, p<0.01).

In contrast, several retrospective studies reported no independent association after adjusting for bacterial contamination and PBD. Tortajada et al.^12^ (2020) and Camps-Lasa et al.^20^ (2024) both observed that fungal contamination did not increase SSI when considered separately from bacteriobilia. Giannone et al.^14^ (2025), also found no direct correlation; however, targeted perioperative antifungal prophylaxis in high-risk patients mitigated the infection burden across both PBD and non-PBD groups.

### Intra-Abdominal Abscess:

Only a limited number of studies evaluated intra-abdominal abscess (iAA) as an independent outcome. Lin et al.^19^ (2021) reported that intraoperative bile contamination, including Candida species, was significantly associated with the development of iAA following PD (OR = 4.994; 95% CI: 1.837–13.572; p = 0.002). However, fungal species were not analyzed separately from bacterial isolates, and the independent effect of fungi could not be determined. Other studies, including Tortajada et al.^12^ and Giannone et al.^14^, did not identify fungal colonization as an isolated risk factor for abscess formation, emphasizing instead the combined impact of polymicrobial bile contamination.

### Clinically Relevant Postoperative Pancreatic Fistula (CR-POPF):

Fungal biliary colonization was inconsistently associated with CR-POPF (Grade B or C). Several studies including Nakamura et al.^21^, Tortajada et al.^12^ and Maatman et al.^15^, reported no significant relationship between fungal colonization and CR-POPF. Meanwhile, Giannone et al.^14^ (2025), observed a significant reduction in CR-POPF rates (p = 0.029) and fewer infectious complications after implementation of a tailored antimicrobial prophylaxis strategy that included antifungals; however, causality could not be confirmed, as fungal involvement was implied rather than directly measured. No study provided multivariable evidence that fungal colonization alone independently predicted CR-POPF.

### Overall Infectious Morbidity and Mortality:

Overall postoperative infectious morbidity tended to be higher in patients with contaminated bile, but the specific effect of fungi remained unclear. Giannone et al.^14^ suggested that adapting perioperative antimicrobial prophylaxis to include antifungal therapy in high-risk patients could reduce postoperative infectious complications. Mortality was infrequently reported. Where available, no study demonstrated increased 30- or 90-day mortality attributed to fungal contamination

### Secondary Outcomes

### Length of Hospital Stay:

Data on length of stay (LOS) relative to fungal colonization were limited. Lin et al.^19^ reported that bile contamination (not exclusively fungi) was associated with prolonged hospitalization, but they did not isolate the effect of Candida. Krueger et al.^18^ also noted longer stays in patients with isolated infectious complications, but the contribution of fungi per se was not clearly delineated. Other studies did not provide fungus-specific LOS data.

### ICU Admission, Reoperation, and Readmission:

These outcomes were rarely reported. Giannone et al.^14^ indicated that patients with fungal colonization had more frequent preoperative ICU admissions (p=0.005), but data on postoperative ICU stay, reoperations, or readmission stratified by fungal status were generally missing or insufficient.

### Antifungal Therapy and Its Impact:

Antifungal therapy was not uniformly used across studies. In most cohorts (e.g., Tortajada, Maatman, Camps-Lasa), antifungals were not part of routine perioperative protocol. Giannone et al.^14^ reported the most robust data: after implementing an antimicrobial regimen that included targeted antifungal therapy, their group observed reductions in CR-POPF, pneumonia, urinary tract infections, and acute respiratory failure. Nonetheless, as this was not a randomized intervention, the precise contribution of antifungal therapy (versus other protocol changes) remains to be confirmed.

### Prevalence and Risk Factors for Fungal Bile Colonization:

Across studies, fungal biliary colonization mostly occurred in patients who underwent PBD. Factors consistently linked with fungal colonization included the presence of a biliary stent, prolonged antibiotic exposure, and higher bilirubin levels. Yang et al.^16^ demonstrated that 90.6% of PBD patients had positive bile cultures compared with 28% of non-PBD patients (p < 0.01), and 4.2% of all PBD patients harbored fungi. Similarly, Camps-Lasa et al.^20^ found Candida in 16.3% of PBD patients versus 0% of non-PBD patients. These findings reinforce the well-described association between stent related microbial colonization and the development of secondary biliary candidiasis.

## DISCUSSION

This systematic review synthesizes the available evidence on fungal biliary colonization in patients undergoing PD. Candida species were the most frequently reported fungi, predominantly in patients with PBD.^12^,^14^,^15^ Across the included studies, fungal colonization rates varied from 2.5% to 43%,^16^,^17^ reflecting differences in sampling methods, microbiological techniques, and patient populations. Despite its relatively frequent occurrence in the setting of biliary manipulation, fungal biliary colonization does not demonstrate a consistent independent association with major postoperative outcomes.

Postoperative infectious morbidity—including SSI, intra-abdominal abscess, and sepsis—was the most commonly evaluated endpoint. A significant association between fungal colonization and SSI,^18^ was reported in two studies, including the sole prospective observational study, which identified biliary candidiasis as an independent risk factor for postoperative infection. In contrast, most retrospective data did not demonstrate an independent association once bacterial contamination, PBD duration, and prior antibiotic exposure were accounted for.^12^,^14^,^16^,^19^,^20^ This discrepancy likely reflects differences in study design, limited sample sizes within fungal subgroups, variability in microbiological techniques, and residual confounding inherent to retrospective analysis. Collectively, these data suggest that fungal colonization more often represents a marker of biliary manipulation and microbial dysbiosis rather than a direct pathogenic driver of postoperative infection. Nevertheless, additional well-designed prospective studies are required to establish definitive conclusions.

With respect to CR-POPF, none of the included studies identified fungal colonization as an independent predictive factor.^21^ One retrospective study reported lower CR-POPF rates^14^ after implementing a prophylactic antimicrobial strategy including antifungal coverage in high-risk PBD patients; however, the bundled intervention precludes attribution of benefit to antifungal therapy alone. Microbiological analyses implicating Enterococcus faecalis—but not fungi—as a predictor of CR-POPF further support the limited mechanistic role of biliary fungi. Postoperative mortality was also unaffected by fungal colonization,^12^,^14^ consistent with the understanding that mortality after PD is primarily driven by severe infections, hemorrhage, or catastrophic POPF.^22^

Secondary outcomes, including length of hospital stay, ICU admission, reoperation, and readmission, were inconsistently reported. When reported, prolonged hospitalization was associated with polymicrobial contamination^18^,^19^ rather than fungal infection alone. Antifungal therapy was used sporadically, with only one study demonstrating improved outcomes with targeted antifungal prophylaxis;^14^ however, concurrent optimization of bacterial coverage complicates interpretation.

Overall, the current evidence indicates that fungal colonization should be considered a surrogate marker of prior biliary instrumentation, prolonged stenting, or antibiotic exposure rather than a primary driver of postoperative morbidity. The heterogeneity of study designs, microbiological methods, and fungal identification techniques limits definitive conclusions. High-quality, multicenter prospective studies with standardized bile sampling, species-level identification, and rigorous adjustment for confounders are needed to clarify the true clinical significance of fungal biliary colonization.

In conclusion, while fungal colonization of bile is common among PBD patients, its independent clinical significance appears limited. Management strategies should focus on optimizing perioperative bacterial coverage and minimizing unnecessary biliary drainage rather than routine antifungal prophylaxis, reserving antifungal therapy for patients with clinical or laboratory evidence of invasive fungal infection.

### Limitations:

This systematic review has several limitations that should be considered when interpreting its findings. Firstly, the number of studies specifically addressing fungal biliary colonization in patients undergoing PD remains limited. Most included studies primarily focused on bacterial contamination of bile, and the identification or analysis of Candida species was often incidental or secondary. Consequently, the data available on the true incidence, species distribution, and clinical impact of fungibilia are incomplete. There is also significant heterogeneity in study design, microbiological methods, and outcome reporting. The timing and technique of bile sampling, as well as the criteria used to define fungal colonization, varied widely between studies. Most studies were retrospective and single-center, which limits external validity and confounding factors such as preoperative biliary drainage (PBD), cholangitis, duration of drainage, and prior antibiotic or antifungal exposure were inconsistently reported or adjusted for. Reporting bias and small sample sizes further constrain interpretation. Only a few studies stratified outcomes by fungal vs. bacterial contamination, and antifungal treatment protocols were rarely standardized or clearly described. Thus, the current literature is insufficient to determine a definitive causal relationship. Future prospective multicenter studies with standardized microbiological and clinical endpoints are needed to clarify the role of fungibilia in PD outcomes and to inform targeted antifungal strategies.

## CONCLUSION

Collectively, available evidence suggests that fungal biliary colonization, predominantly Candida species, is common in patients with preoperative biliary drainage and may contribute to an increased risk of postoperative infectious complications after pancreaticoduodenectomy. However, this association appears largely mediated by concurrent bacterial contamination and the presence of biliary stents. No high-quality evidence demonstrates a direct, independent causal relationship between fungal colonization and postoperative morbidity or mortality. Adapted antimicrobial prophylaxis including antifungal coverage may offer benefits in selected high-risk patients, but randomized controlled trials are lacking.

### Authors Contribution:

**NM** contributed to the conception, design and drafting of the manuscript.

**NM and RA** were responsible for the methodology, literature search, data screening and extraction and manuscript writing.

**SB and MRK:** provided supervision and contributed to critical review and editing of the manuscript.

All authors have approved the final version of the manuscript, and agree to be accoun for all aspects of the work.

## Appendix

**Section 1:** PRISMA Checklist (2020)

Section and Topic	Item #	Checklist item	Location where item is reported
**TITLE**			
Title	1	Identify the report as a systematic review.	Page 1
**ABSTRACT**			
Abstract	2	See the PRISMA 2020 for Abstracts checklist.	Page 1
**INTRODUCTION**			
Rationale	3	Describe the rationale for the review in the context of existing knowledge.	Page 2-3
Objectives	4	Provide an explicit statement of the objective(s) or question(s) the review addresses.	Page 3
**METHODS**			
Eligibility criteria	5	Specify the inclusion and exclusion criteria for the review and how studies were grouped for the syntheses.	Page 3
Information sources	6	Specify all databases, registers, websites, organisations, reference lists and other sources searched or consulted to identify studies. Specify the date when each source was last searched or consulted.	Page 3
Search strategy	7	Present the full search strategies for all databases, registers and websites, including any filters and limits used.	Supplementary section 2
Selection process	8	Specify the methods used to decide whether a study met the inclusion criteria of the review, including how many reviewers screened each record and each report retrieved, whether they worked independently, and if applicable, details of automation tools used in the process.	Page 3
Data collection process	9	Specify the methods used to collect data from reports, including how many reviewers collected data from each report, whether they worked independently, any processes for obtaining or confirming data from study investigators, and if applicable, details of automation tools used in the process.	Page 3-4
Data items	10a	List and define all outcomes for which data were sought. Specify whether all results that were compatible with each outcome domain in each study were sought (e.g. for all measures, time points, analyses), and if not, the methods used to decide which results to collect.	Page 4
	10b	List and define all other variables for which data were sought (e.g. participant and intervention characteristics, funding sources). Describe any assumptions made about any missing or unclear information.	Page 3-4
Study risk of bias assessment	11	Specify the methods used to assess risk of bias in the included studies, including details of the tool(s) used, how many reviewers assessed each study and whether they worked independently, and if applicable, details of automation tools used in the process.	Page 4
Effect measures	12	Specify for each outcome the effect measure(s) (e.g. risk ratio, mean difference) used in the synthesis or presentation of results.	Page 4
Synthesis methods	13a	Describe the processes used to decide which studies were eligible for each synthesis (e.g. tabulating the study intervention characteristics and comparing against the planned groups for each synthesis (item #5)).	Page 3
	13b	Describe any methods required to prepare the data for presentation or synthesis, such as handling of missing summary statistics, or data conversions.	N/A
	13c	Describe any methods used to tabulate or visually display results of individual studies and syntheses.	Page 4
	13d	Describe any methods used to synthesize results and provide a rationale for the choice(s). If meta-analysis was performed, describe the model(s), method(s) to identify the presence and extent of statistical heterogeneity, and software package(s) used.	Page 4
	13e	Describe any methods used to explore possible causes of heterogeneity among study results (e.g. subgroup analysis, meta-regression).	N/A
	13f	Describe any sensitivity analyses conducted to assess robustness of the synthesized results.	N/A
Reporting bias assessment	14	Describe any methods used to assess risk of bias due to missing results in a synthesis (arising from reporting biases).	Page 4
Certainty assessment	15	Describe any methods used to assess certainty (or confidence) in the body of evidence for an outcome.	N/A
**RESULTS**			
Study selection	16a	Describe the results of the search and selection process, from the number of records identified in the search to the number of studies included in the review, ideally using a flow diagram.	Page 4
	16b	Cite studies that might appear to meet the inclusion criteria, but which were excluded, and explain why they were excluded.	N/A
Study characteristics	17	Cite each included study and present its characteristics.	Page 4-6
Risk of bias in studies	18	Present assessments of risk of bias for each included study.	Page 10
Results of individual studies	19	For all outcomes, present, for each study: (a) summary statistics for each group (where appropriate) and (b) an effect estimate and its precision (e.g. confidence/credible interval), ideally using structured tables or plots.	Page 11-12
Results of syntheses	20a	For each synthesis, briefly summarise the characteristics and risk of bias among contributing studies.	Page 10
	20b	Present results of all statistical syntheses conducted. If meta-analysis was done, present for each the summary estimate and its precision (e.g. confidence/credible interval) and measures of statistical heterogeneity. If comparing groups, describe the direction of the effect.	N/A
	20c	Present results of all investigations of possible causes of heterogeneity among study results.	N/A
	20d	Present results of all sensitivity analyses conducted to assess the robustness of the synthesized results.	N/A
Reporting biases	21	Present assessments of risk of bias due to missing results (arising from reporting biases) for each synthesis assessed.	N/A
Certainty of evidence	22	Present assessments of certainty (or confidence) in the body of evidence for each outcome assessed.	N/A
**DISCUSSION**			
Discussion	23a	Provide a general interpretation of the results in the context of other evidence.	Page 6-8
	23b	Discuss any limitations of the evidence included in the review.	Page 8
	23c	Discuss any limitations of the review processes used.	Page 8
	23d	Discuss implications of the results for practice, policy, and future research.	Page 8
**OTHER INFORMATION**			
Registration and protocol	24a	Provide registration information for the review, including register name and registration number, or state that the review was not registered.	Page 3
	24b	Indicate where the review protocol can be accessed, or state that a protocol was not prepared.	Page 3
	24c	Describe and explain any amendments to information provided at registration or in the protocol.	N/A
Support	25	Describe sources of financial or non-financial support for the review, and the role of the funders or sponsors in the review.	8
Competing interests	26	Declare any competing interests of review authors.	8
Availability of data, code and other materials	27	Report which of the following are publicly available and where they can be found: template data collection forms; data extracted from included studies; data used for all analyses; analytic code; any other materials used in the review.	3

***From:*** Page MJ, McKenzie JE, Bossuyt PM, Boutron I, Hoffmann TC, Mulrow CD, et al. The PRISMA 2020 statement: an updated guideline for reporting systematic reviews.

BMJ 2021;372:n71. doi: 10.1136/bmj.n71

**Section 2: Search Strings** (searched on 15^th^ October, 2025).
**PubMed:**
((“Fungi”[Mesh] OR “Mycoses”[Mesh] OR fungal OR fungi OR candida OR candidiasis OR “Candida albicans”) AND (“Bile”[Mesh] OR “Biliary Tract”[Mesh] OR Biliary Drainage OR biliary OR “biliary stent*” OR “bile duct*” OR “bile contamination” OR “bile colonization”) AND (“Pancreaticoduodenectomy”[Mesh] OR “Whipple” OR “pancreatic resection” OR “pancreatic head resection” OR PD) AND (“Postoperative Complications”[Mesh] OR “Surgical Site Infection” OR “morbidity” OR “mortality” OR “outcome*” OR postoperative pancreatic fistula OR delayed gastric emptying OR postpancreatectomy hemorrhage))
**Scopus:**
((“Fungi” OR “Mycoses” OR fungal OR fungi OR candida OR candidiasis OR “Candida albicans”) AND (“Bile” OR “Biliary Tract” OR Biliary Drainage OR biliary OR “biliary stent” OR “bile duct” OR “bile contamination” OR “bile colonization”) AND (“Pancreaticoduodenectomy” OR “Whipple” OR “pancreatic resection” OR “pancreatic head resection” OR PD) AND (“Postoperative Complications” OR “Surgical Site Infection” OR “morbidity” OR “mortality” OR “outcome” OR ‘postoperative pancreatic fistula’ OR ‘delayed gastric emptying’ OR ‘postpancreatectomy hemorrhage’))
**Embase:**
(’mycoses’/exp OR ‘mycoses’ OR ‘fungal’ OR fungi OR ‘candida’/exp OR ‘candidiasis’/exp OR ‘candida albicans’/exp) AND (’bile’/exp OR ‘biliary tract’/exp OR ‘biliary drainage’/exp OR biliary OR ‘biliary stent’/exp OR ‘bile duct’/exp OR ‘bile contamination’ OR ‘bile colonization’) AND (’pancreaticoduodenectomy’/exp OR ‘whipple’ OR ‘pancreatic resection’/exp OR ‘pancreatic head resection’/exp OR ‘pd’/exp) AND (’postoperative complications’/exp OR ‘surgical site infection’/exp OR ‘morbidity’/exp OR ‘mortality’/exp OR ‘outcome’/exp OR ‘postoperative pancreatic fistula’/exp OR ‘delayed gastric emptying’/exp OR ‘postpancreatectomy hemorrhage’/exp)
**Cochrane:**
#1: MeSH descriptor: [Fungi] explode all trees #2: MeSH descriptor: [Mycoses] explode all trees #3: (fungal OR fungi OR candida OR candidiasis OR “Candida albicans”) #4: #1 OR #2 OR #3 #5: MeSH descriptor: [Bile] explode all trees #6: MeSH descriptor: [Biliary Tract] explode all trees #7: (Biliary Drainage OR biliary OR “biliary stent” OR “bile duct” OR “bile contamination” OR “bile colonization”) #8: #5 OR #6 OR #7 #9: MeSH descriptor: [Pancreaticoduodenectomy] explode all trees #10: (“Whipple” OR “pancreatic resection” OR “pancreatic head resection” OR PD) #11: #9 OR #10 #12: MeSH descriptor: [Postoperative Complications] explode all trees #13: (“Surgical Site Infection” OR “morbidity” OR “mortality” OR “outcome” OR “postoperative pancreatic fistula” OR “delayed gastric emptying” OR “postpancreatectomy hemorrhage”) #14: #12 OR #13 #15: #4 AND #8 AND #11 AND #14
